# Supplementary material for: The interactome of intact mitochondria by cross-linking mass spectrometry provides evidence for coexisting respiratory supercomplexes
Source: Mol Cell Proteomics. 2017 Dec 8;17(2):216–32. doi: 10.1074/mcp.RA117.000470 (PMC5795388; doi:10.1074/mcp.RA117.000470)
Supplement: Supplemental Data [file supp_RA117.000470_133922_0_supp_23978_fzffwf.zip › spectra_annotation/mito_spectra_annotation/76-1-8-1-33-1.pdf]

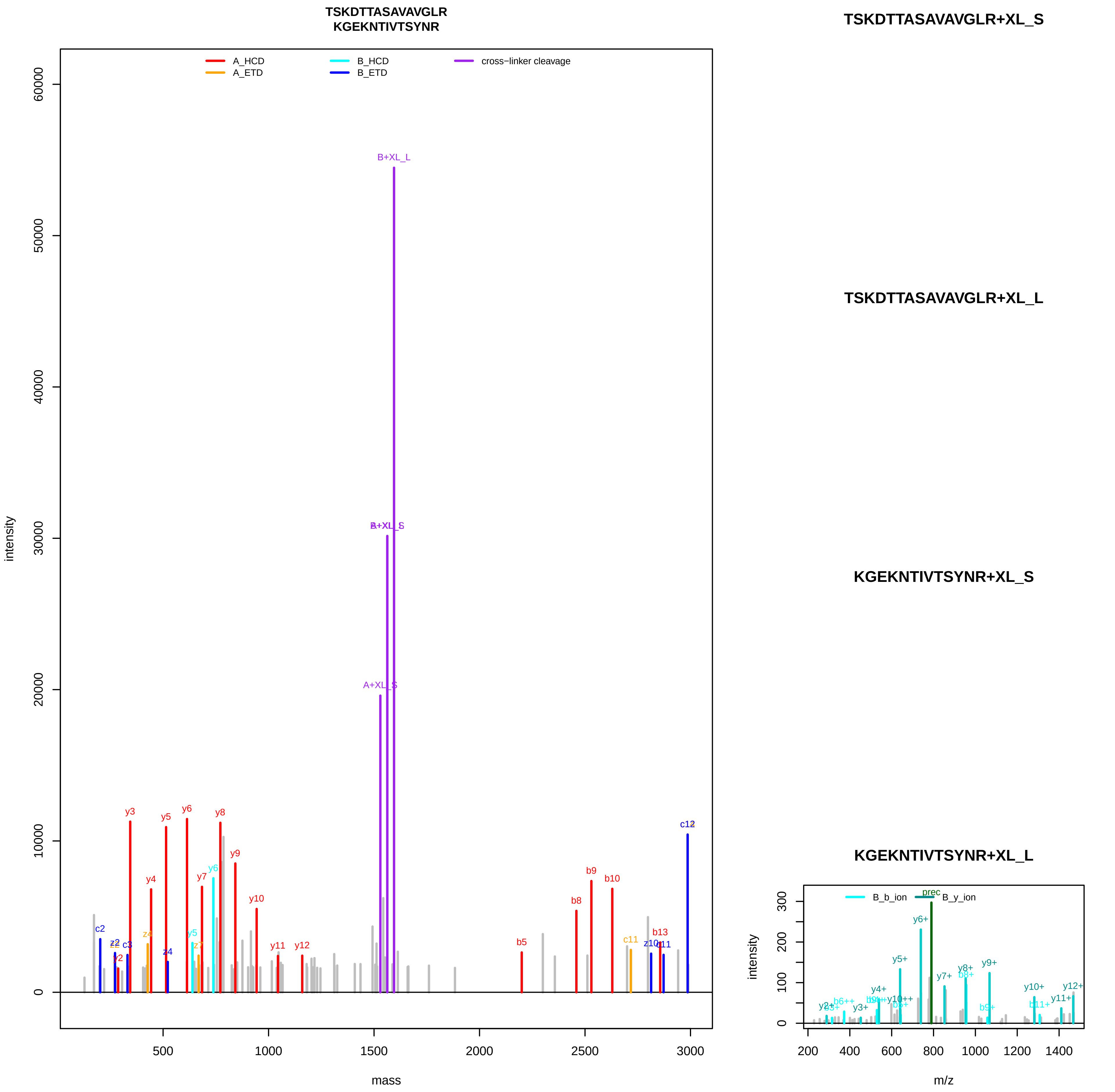

TSKDTTASAVAVGLR
KGEKNTIVTSYNR
A_HCD
A_ETD
B_HCD
B_ETD
cross−linker cleavage
B+XL_L
A+XL_S
intensity
mass
0
10000
20000
30000
40000
50000
60000
500
1000
1500
2000
2500
3000
y3
y4
y5
y6
y7
y8
y9
y10
y11
y12
b5
b8
b9
b10
b13
c2
c3
z2
z4
c11
c12
z10
TSKDTTASAVAVGLR+XL_S
TSKDTTASAVAVGLR+XL_L
KGEKNTIVTSYNR+XL_S
KGEKNTIVTSYNR+XL_L
B_b_ion
B_y_ion
prec
y5+
y6+
y7+
y8+
y9+
y10+
y11+
y12+
y4+
y3+
y2+
b9+
b11+
m/z
200
400
600
800
1000
1200
1400
0
100
200
300
